# Interfacial interaction driven enhancement in the colossal magnetoresistance property of ultra-thin heterostructure of Pr_0.6_Sr_0.4_MnO_3_ in proximity with Pr_0.5_Ca_0.5_MnO_3_

**DOI:** 10.1038/s41598-023-28314-8

**Published:** 2023-02-09

**Authors:** V. Gayathri, E. P. Amaladass, A. T. Sathyanarayana, T. Geetha Kumary, R. Pandian, Pooja Gupta, Sanjay K. Rai, Awadhesh Mani

**Affiliations:** 1 Materials Science Group, Indira Gandhi Centre for Atomic Research, Kalpakkam, 603102 India; 2 Homi Bhabha National Institute, Training School Complex, Anushaktinagar, Mumbai, 400094 India; 3 Accelerator Physics and Synchrotrons Utilisation Division, Raja Ramanna Centre for Advanced Technology, Indore, 452013 India

**Keywords:** Materials science, Nanoscience and technology, Physics

## Abstract

The ultra-thin heterostructure of Pr_0.6_Sr_0.4_MnO_3_(15 nm)/Pr_0.5_Ca_0.5_MnO_3_(15 nm)/SrTiO_3_ fabricated using pulsed laser deposition technique exhibits the phase-segregated nature wherein the ferromagnetism of Pr_0.6_Sr_0.4_MnO_3_, and the antiferromagnetic state of Pr_0.5_Ca_0.5_MnO_3_ coexist in proximity. The observation of two exciting phenomena in the grown ultra-thin heterostructure, namely, the kinetic arrest and training effect, confirms its phase-segregated nature. The melting of the antiferromagnetic state in Pr_0.5_Ca_0.5_MnO_3_ into a ferromagnetic state due to the interfacial interaction arising from the magnetic proximity of the ferromagnetic clusters of Pr_0.6_Sr_0.4_MnO_3_ have been observed. A metal–insulator transition (T_MIT_) found at 215 K, close to its Curie temperature (T_Curie_) observed at 230 K, reveals a strong correlation between the electrical transport and the magnetization of the ultra-thin heterostructure. The electrical conduction in the high-temperature regime is explained in terms of the adiabatic small polaron hopping model. While the resistance in the metallic regime for temperatures above 100 K is contributed by the inelastic scattering due to the two-magnons, in the metallic regime below 100 K, the one-magnon inelastic scattering contribution is prevalent. An enhanced colossal magnetoresistance property near room temperature is obtained in the ultra-thin heterostructure arising from the proximity-driven interfacial interaction, making it a suitable candidate for technological applications near room temperature.

## Introduction

Perovskite manganites represented by the general formula, R_1−x_A_x_MnO_3_ (R = Rare earth ion, A = Alkaline earth ion) being strongly correlated electron systems have emerged as exciting material for spintronics applications due to its fascinating structural, electronic, and magnetic properties^1,2^. Several interesting phenomena like colossal magnetoresistance, diverse charge, spin, and orbital orderings, phase-segregation related kinetic arrest and training effect, and metal–insulator transitions can be realized in these materials by local entanglement of the charge, spin, and orbital degrees of freedom due to their inherent electron correlation induced constraint in the number of electrons at a given lattice site^3–6^. The colossal magnetoresistance (CMR) in manganites is usually observed in the doping range of 0.2 < x ≤ 0.5 and is characterized by a considerable fall in the resistance of a material as a response to the applied magnetic field^4^. The origin of the CMR can be understood on the basis of the Zener double exchange mechanism between the Mn^3+^ and Mn^4+^ ions, the Jahn–Teller effect, and the nanoscale electronic phase segregation related intrinsic inhomogeneities present in these manganites^7–9^. The research on manganites continues to attract interest due to the diversity of their physical properties and their potential for usage in technological applications. The majority of device application requires the materials to be harnessed in the thin-film form due to their perpetual miniaturization into nanoscale. The pulsed laser deposition (PLD) technique is one of the most widely used techniques to fabricate thin films^10,11^. The ability to deposit the materials in a reactive environment makes PLD a suitable technique for fabricating thin films of oxide materials like manganites. However, the sensitive nature of the manganites towards any external perturbation like strain or lattice distortion alters their physical properties in the thin-film form, making it yet another exciting topic of research interest^1,2,12,13^.

The single crystals of Pr_0.6_Sr_0.4_MnO_3_ (PSMO) undergo a paramagnetic (PM) to ferromagnetic (FM) transition^12,14,15^ (T_Curie_ ~ 315 K) with a concomitant insulator to metal transition (T_MIT_)^15^ near room temperature, thereby exhibiting a strong correlation between the electrical and magnetic properties. Nonetheless, in the polycrystalline PSMO, the T_MIT_ occurs at temperatures lower than its T_Curie_^12,16,17^. Large CMR effects near its T_MIT_ have been reported in the PSMO thin films deposited on various substrates compared to the bulk sample^12^. However, the T_MIT_ in the PSMO thin film of ~ 30 nm occurs at a lower temperature ~ 85 K, than the bulk, with a maximum CMR of ~ 90% at 5 T^12^. The half-doped manganite, Pr_0.5_Ca_0.5_MnO_3_ (PCMO), has been reported to undergo a phase transition from the high temperature paramagnetic (PM) insulating state to a charge-ordered (CO) insulating state below ~ 250 K, followed by a transition into a CE-type antiferromagnetic (AFM) state below ~ 170 K^18^ in its bulk form. This CE-AFM state of PCMO is described by the zig-zag ferromagnetic chains in the *a*−*b* plane aligned antiferromagnetically in all the other directions^19^. A huge CMR effect can be realized in PCMO by melting the AFM state into the FM state by the application of the magnetic field^20,21^. However, the requirement of a large melting field (~ 25 T) hinders its potential for device applications^21^. With the development of thin-film fabrication techniques like the PLD technique, the strain-induced lowering of the melting field of the AFM state has been reported in the PCMO thin films^13,22,23^. Nevertheless, the T_MIT_ and huge CMR effects are reported in PCMO thin films at lower temperatures. The feasibility of using these materials for device fabrication necessitates achieving large values of low field CMR and T_MIT_ near room temperature. The interfacial interactions of two competing ground states (FM metallic and CO insulating states) in the bilayered/multilayered heterostructures of two manganites with different physical properties have been reported to enhance the CMR effect^24–28^. Our previous report on heterostructural bilayer film of Pr_0.6_Sr_0.4_MnO_3_ and Pr_0.5_Ca_0.5_MnO_3_ with each layer having a thickness of 300 nm exhibited an enhancement in the CMR property near room temperature^29^.

In the present work, PSMO(15 nm)/PCMO(15 nm)/STO ultra-thin heterostructure was fabricated using the PLD technique. The grown 30 nm ultra-thin heterostructure comprising 15 nm PSMO on top of 15 nm PCMO deposited on the STO substrate is addressed as PSMO/PCMO/STO in the following text. The heterostructure was characterized for its structural, morphological, magnetotransport, and magnetization properties. The present study explores the suitability of using PSMO/PCMO/STO ultra-thin heterostructure for colossal magnetoresistance applications near room temperature.

## Results and discussion

### Structural analyses

**Figure 1:**
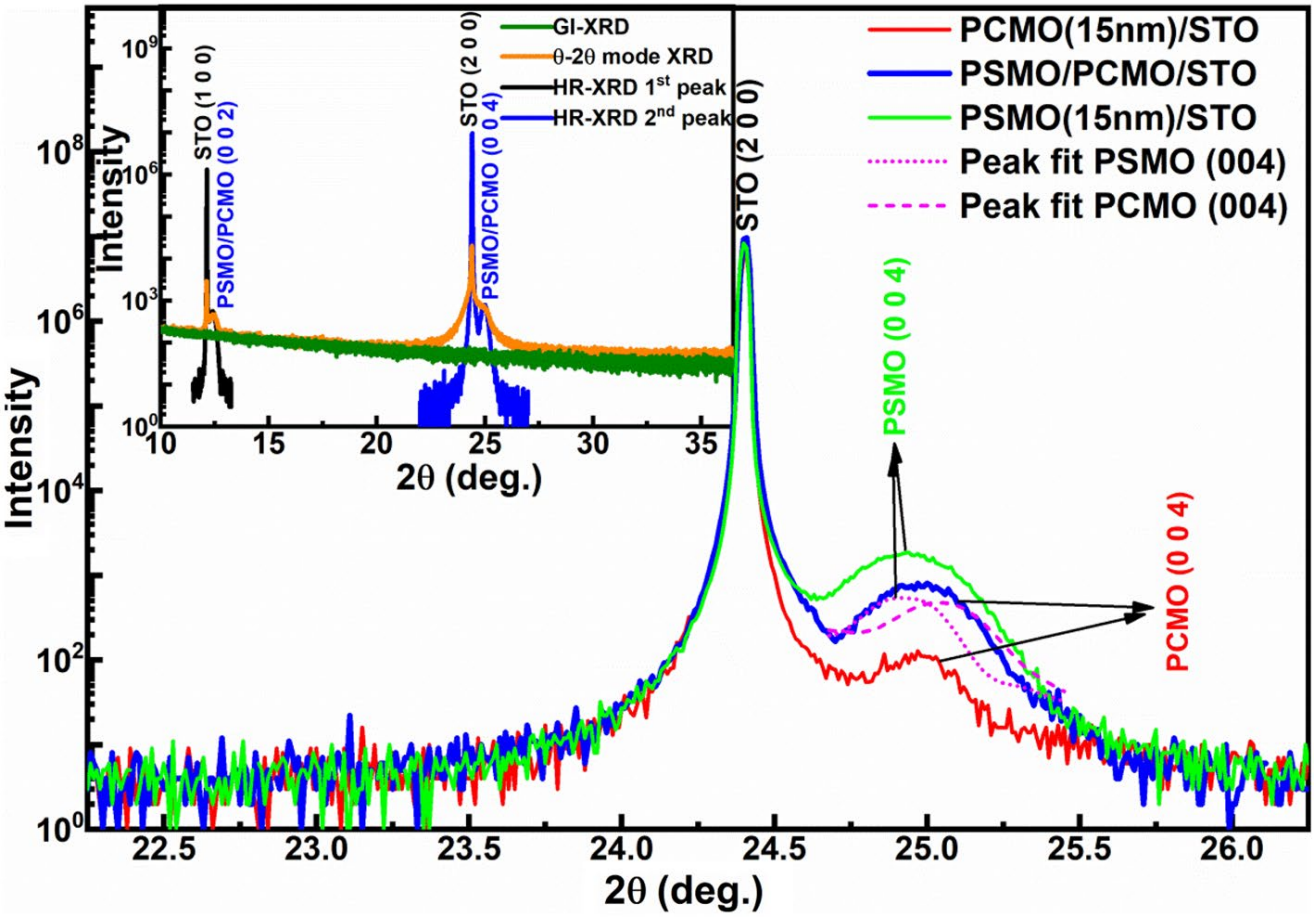
HR-XRD of PSMO/PCMO/STO heterostructure near (2 0 0) reflection of STO. Inset shows the XRD pattern obtained in GI, θ−2θ, and HR modes for the heterostructure.

The inset in Fig. 1 shows the X-ray diffraction (XRD) pattern obtained for the PSMO/PCMO/STO heterostructural thin film in grazing-incidence (GI), θ−2θ, and high-resolution (HR) modes. The XRD performed in the θ−2θ mode revealed the presence of *(0 0 2 l)* reflections from the sample close to the *(1 0 0)* oriented substrate peaks. Further, HR-XRD was performed near the *(1 0 0)* and *(2 0 0)* reflections of STO to obtain distinct reflections from the heterostructure. Figure 1 illustrates the HR-XRD of PSMO/PCMO/STO heterostructure near the *(2 0 0)* reflection of STO (located at 24.4°) in the 2θ range of 22–27°. A broad peak pertaining to the heterostructure was obtained near the *(2 0 0)* reflection of STO. Upon deconvolution of this broad peak using pseudo voigt function, *(0 0 4)* reflections of PSMO and PCMO were obtained at 24.93° and 25.05°, respectively. The peaks were indexed by comparing them with the standard ICDD data^30,31^. These deconvoluted peak positions match with the corresponding peak positions of their single layers of similar thicknesses, as shown in Fig. 1. The presence of *(0 0 2 l)* peaks of the grown heterostructure confirms the *c*-axis orientation of the films. The absence of reflections from the sample in the GI-XRD, as shown in the inset of Fig. 1, further confirms the growth direction of the heterostructural film to be preferentially *c*-axis. The *c*-lattice parameters as calculated from the inter-planar spacing using Bragg’s law are found to be 7.62 Å and 7.65 Å, for the PCMO and PSMO layer in the heterostructure, respectively, which are akin to their annealed single layer counterparts. Hence, the presence of both layers in the heterostructure is confirmed.

### Morphological analyses

**Figure 2:**
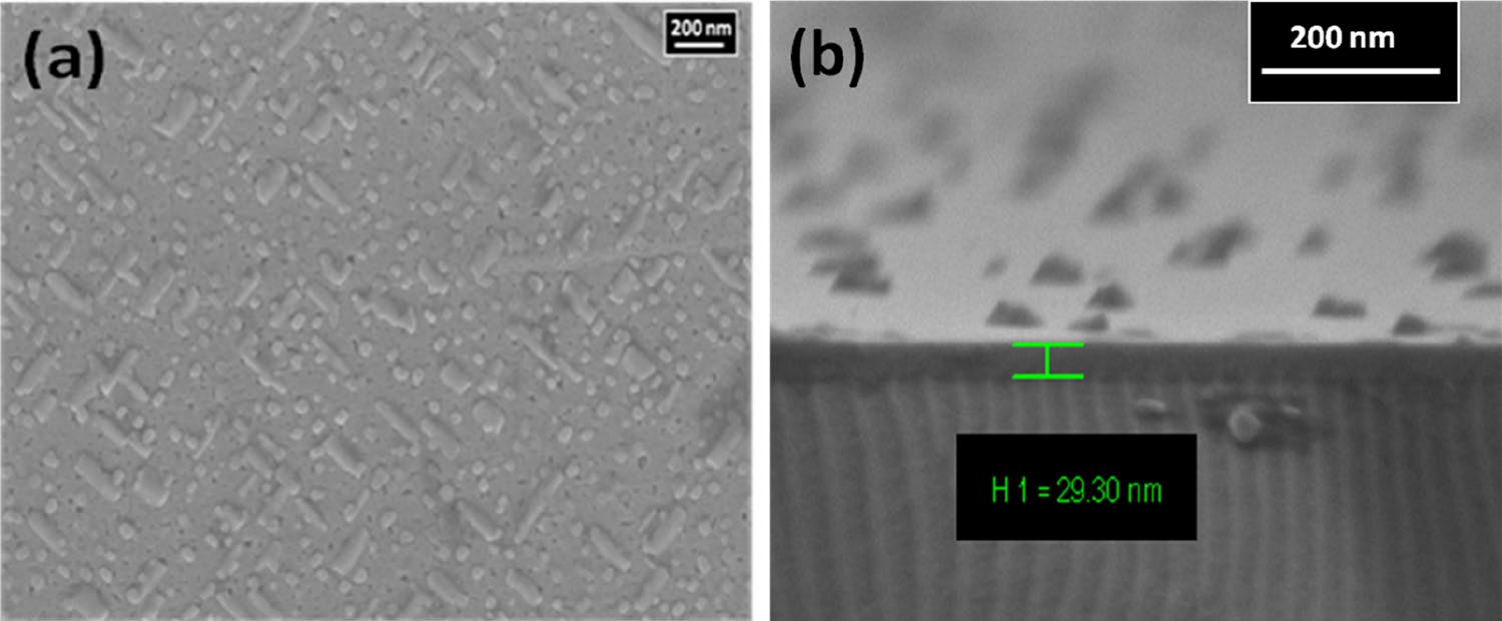
(**a**) Plan view SEM of PSMO/PCMO/STO heterostructure showing the crossed rod-shaped morphology; (**b**) Cross-sectional SEM of the heterostructure illustrating the total thickness of the heterostructure to be 30 nm.

**Figure 3:**
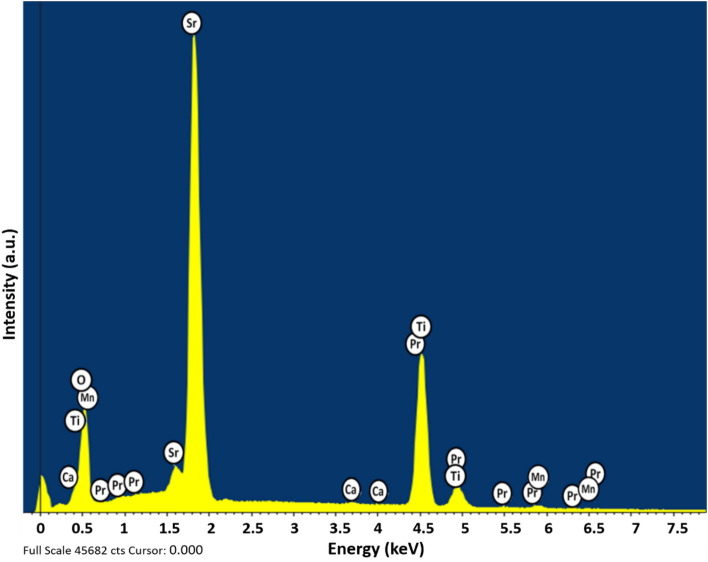
EDS spectrum obtained for PSMO/PCMO/STO heterostructure.

Morphology and the thickness of the grown PSMO/PCMO/STO heterostructure were revealed using the plan-view and the cross-sectional view mode of the scanning electron microscopy (SEM). Figure 2a represents the SEM micrograph of the grown PSMO/PCMO/STO heterostructure in the plan-view mode. The micrograph shows the presence of rod-shaped grains embedded in a smooth matrix of the heterostructure. These grains are nearly a few hundreds of nm long and a few tens of nm wide. Figure 2b illustrates the determination of the thickness of the ultra-thin heterostructure using the cross-sectional mode of the SEM. The total thickness of the heterostructure, as determined from the cross-sectional mode of the SEM, was found to be ~ 30 nm. The elemental analysis using energy-dispersive X-ray spectroscopy (EDS) confirmed the presence of all the elements in the heterostructure without any impurity as shown in Fig. 3.

### Magnetization studies

**Figure 4:**
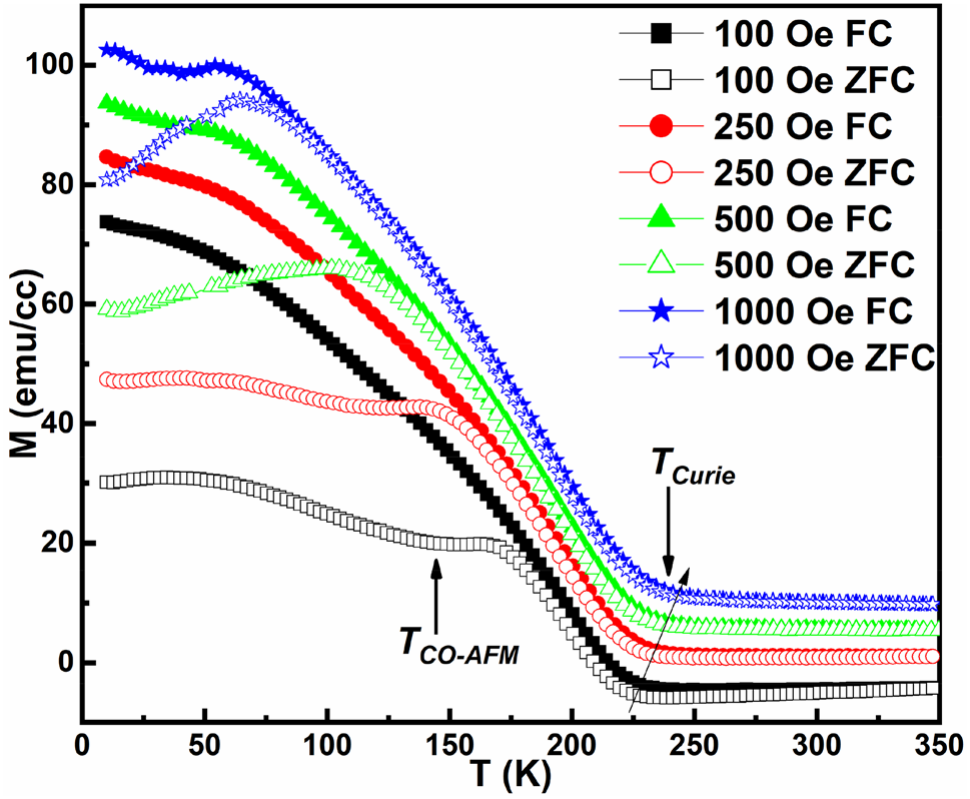
Temperature-dependent magnetization measurement at various applied magnetic fields for PSMO/PCMO/STO heterostructure.

The temperature-dependent magnetization plot (M(T)) obtained for PSMO/PCMO/STO heterostructure is shown in Fig. 4. Field cooled (FC) and zero field cooled (ZFC) data are shown by solid and hollow symbols, respectively. FC and ZFC curves were obtained for values of the magnetic field in the range of 100–1000 Oe applied parallel to the plane of the heterostructure. The Curie temperature (T_Curie_) is determined from the M(T) curve using the method of double-tangent intersection point. From the M(T) curves, it is clearly discernable that the heterostructure undergoes a paramagnetic (PM) to ferromagnetic (FM) transition at T_Curie_ ~ 230 K for an applied magnetic field of 100 Oe. The value of T_Curie_ is found to shift to higher temperature with an increase in the value of the applied magnetic field. However, the value of T_Curie_ obtained for PSMO/PCMO/STO is found to be lower than that obtained for the bulk PSMO (T_Curie_ = 315 K)^12^. A second transition observed at around 150 K, as indicated by an arrow in Fig. 4 for H = 100 Oe, can be attributed to the magnetic transition of PCMO from the charge-ordered (CO) to the antiferromagnetic (AFM) state. This transition (T_CO-AFM_) is found to shift to lower temperatures with an increase in the value of H. This field-dependent shift towards lower temperature may be due to the magnetic proximity effect related enhancement in the magnetic exchange interaction resulting in a competition between the AFM state of PCMO and the FM state of PSMO. Since the ferromagnetism of PSMO gets strengthened with the field, it may suppress the antiferromagnetism of PCMO by shifting it to lower temperatures or eventually melting it into the FM state. These can be the signatures of the proximity of PSMO on PCMO, which become prominent in the ultra-thin heterostructures.

The ZFC and the FC curves were found to diverge below the irreversibility temperature with the ZFC curve running below the FC curve. In samples containing randomly oriented nano-crystallites, a divergence in the ZFC and FC curves is expected^16^. The ultra-thin heterostructure comprises nano-grains, as inferred from SEM micrographs. The observed magnetization behaviour can be explained qualitatively by considering the competition between the magneto-crystalline anisotropy energy and the Zeeman energy in these crystallites^29^. This divergence of ZFC and FC curves shows that all the domains are not aligned in the ultra-thin heterostructure. However, the magnetic moment of the ZFC curve at low temperatures is not negligible, as can be seen from the values of the magnetization (M) in the ZFC condition. The obtained M value of 30 emu/cc at 10 K in the ZFC curve of 100 Oe, shows the presence of aligned domains in the ultra-thin heterostructure.

**Figure 5:**
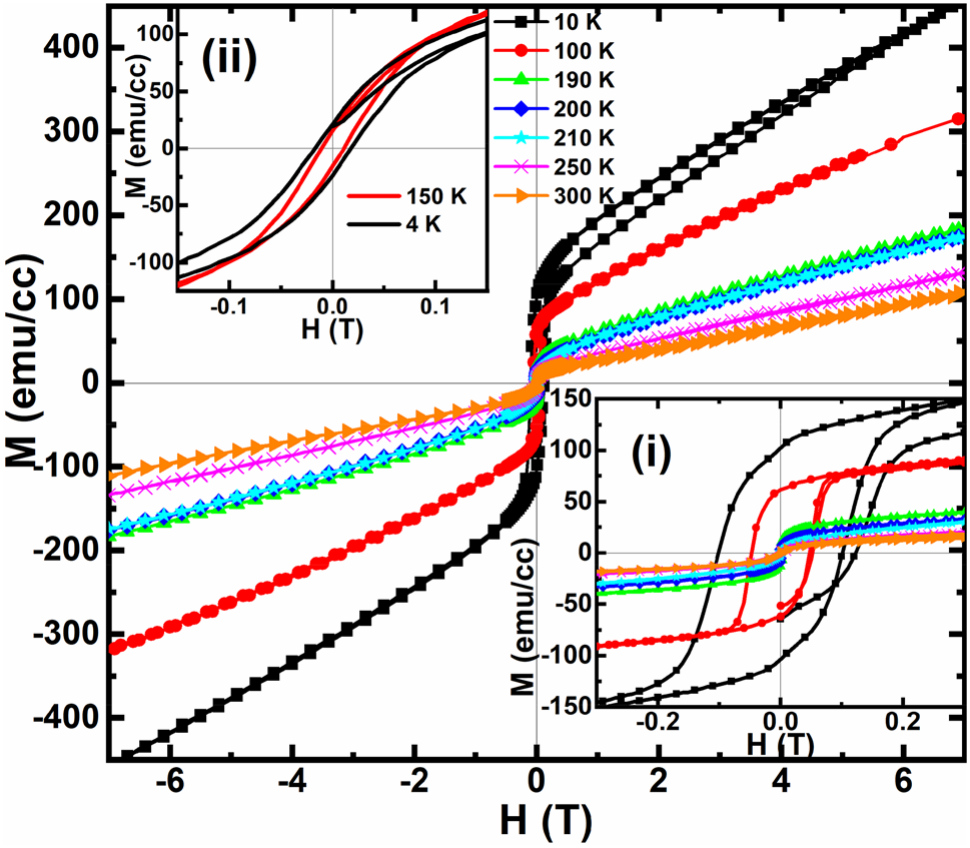
Magnetic field-dependent magnetization measurement at various temperatures for PSMO/PCMO/STO heterostructure. Insets (i) and (ii) show the enlarged view of the hysteresis loop obtained for the PSMO/PCMO/STO heterostructure and the PCMO(15 nm)/STO thin film.

The magnetic field-dependent magnetization (M-H) curve for PSMO/PCMO/STO heterostructure at various temperatures is shown in Fig. 5. For 190 K ≤ T ≤ 300 K, the M-H curve exhibits predominantly PM behaviour with unsaturated magnetic moments. For T = 190 K, 200 K, and 210 K, the hysteresis is negligible since the temperature range is close to its T_Curie_. It is also observed that the magnetic saturation is not reached even under an applied field of 7 T. The absence of magnetic saturation even up to 7 T confirms the presence of antiferromagnetism coexisting in the heterostructure, which may be arising from the AFM state of the PCMO, which failed to get annihilated and is persisting even at high temperatures^29^. The heterostructure exhibits a symmetric hysteresis loop at low temperatures for T = 10 K and 100 K, and the coercive field is found to increase with the decrease in the temperature as evident from the inset (i) of Fig. 5. Furthermore, the value of the remnant magnetization is observed to increase with decreasing temperature in the ultra-thin heterostructure. The value of the remnant magnetization at 10 K is found to be ~ 100 emu/cc. The increase in the remnant magnetization and the coercive field indicates the increase in the strength of the FM phase in the heterostructure. These observations can be understood by considering the following points. The presence of the strain-induced uncompensated spins in the ultra-thin PCMO layer as revealed from the M-H curve of the 15 nm thin film of PCMO grown on STO substrate shown in the inset (ii) of Fig. 5, can contribute to the FM of the heterostructure^32^. Additionally, at temperatures below 150 K, the PCMO enters into the AFM state. With the application of the magnetic field, this AFM state can melt into the FM state. The proximity of the ferromagnetic PSMO can also induce ferromagnetism in a few interfacial layers of PCMO, which can further trigger the melting of the AFM state of PCMO into the FM state. In a nutshell, in the heterostructure, the fraction of the FM phase has been remarkably enhanced due to the presence of the uncompensated spins in the ultra-thin PCMO film and the proximity-driven magnetic transition of the AFM state in PCMO into the FM state. Thus, both PSMO and PCMO contribute to the ferromagnetism exhibited by the heterostructure at low temperatures. The hysteresis loop has a pronounced unsaturated magnetic moment even at higher fields. The observed pronounced unsaturating magnetic moments at low temperatures can be due to the presence of some of the AFM states coexisting with the FM states, which escaped the effect of the proximity of PSMO and remained to be AFM.

### Magnetotransport studies (R(T, H))

**Figure 6:**
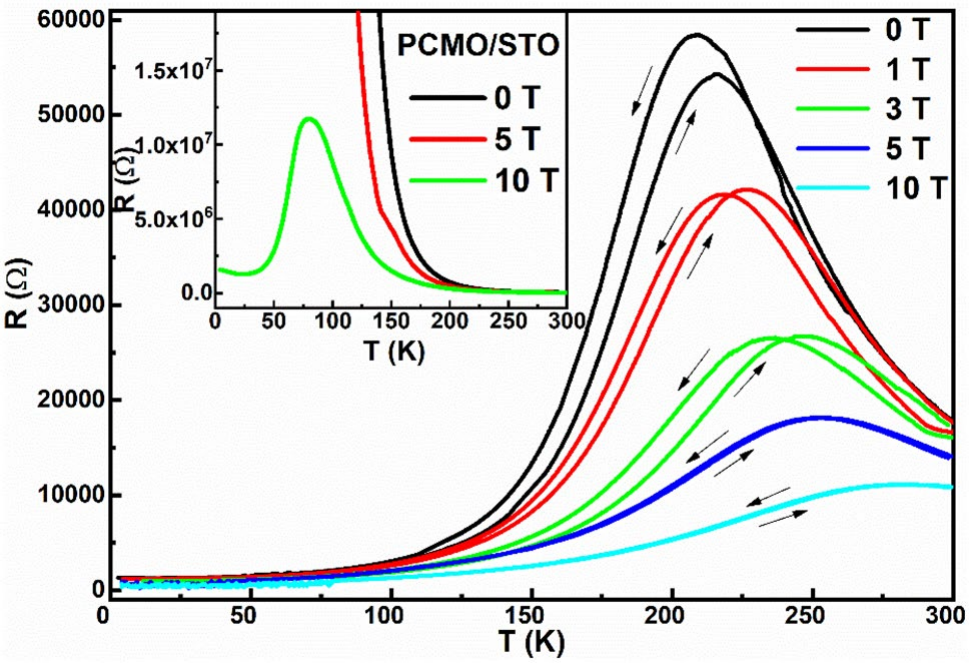
Temperature-dependent variation of resistance for various applied magnetic fields obtained for both cooling and warming cycles of PSMO/PCMO/STO heterostructure. Inset shows the temperature-dependent variation of resistance of PCMO/STO thin film.

The temperature-dependent variation in the resistance (R-T) of the grown PSMO/PCMO/STO heterostructure for both cooling and warming cycles as a function of the applied magnetic field is presented in Fig. 6. The heterostructure exhibits a metal–insulator transition at T_MIT_ = 215 K at 0 T. A thermal hysteresis between the cooling and warming cycles in the metallic and the insulating branches is observed. In the warming cycle, the T_MIT_ is found to exhibit a small shift towards higher temperatures. It is speculated that the observed shift of T_MIT_ is due to the increase in the volume of the metallic FM phase in the warming cycle. The thermal hysteresis shows the presence of phase segregation in the heterostructure. With the application of the magnetic field, the T_MIT_ is found to shift to higher temperatures accompanied by a huge decrease in the resistance and a decrease in the area of the thermal hysteresis in the magnetotransport curves. The R-T measurements of the PCMO thin film grown on STO substrate is illustrated in the inset of Fig. 6. The values of T_MIT_ at 0 T, 5 T, and 10 T obtained for the ultra-thin PSMO/PCMO/STO heterostructure is compared with that of its thicker heterostructure PSMO(300 nm)/PCMO(300 nm)/STO, the PSMO/STO single layer, the PSMO bulk, and the PCMO/STO thin film, and are tabulated in Table 1. The R-T curve of the PCMO/STO as shown in the inset of Fig. 6 reveals that the PCMO thin film is insulating for applied magnetic field (H) values of H = 0 T and 5 T. At H = 10 T, the insulating CO-AFM state of PCMO was found to melt into a ferromagnetic metallic state at T ~ 80 K. Thus, for H < 10 T, PCMO being in the insulating state, is not expected to contribute to the transport properties. T_MIT_ is found to shift from 215 K at H = 0 T to 280 K at H = 10 T in the ultra-thin PSMO/PCMO/STO heterostructure. From Table 1, it is evident that these shifts are more prominent in the ultra-thin heterostructure than in the thicker heterostructure^29^, the single layer thin film and the bulk^12^. Thus, the signatures of proximity effect can be perceived in the ultra-thin heterostructure, wherein a magnetic field-induced increase in the FM metallic phase in PSMO can trigger electrical conduction in the interfacial layers of PCMO due to the magnetic proximity effect, thereby shifting the T_MIT_ at higher fields to near room temperatures. Additionally, it is quite interesting to note that, in the present study, the values of T_Curie_ and T_MIT_ are closely associated.

**Table 1:** Comparison of the T_MIT_ at 0 T, 5 T, and 10 T of ultra-thin PSMO/PCMO/STO heterostructure with the thicker heterostructure PSMO(300 nm)/PCMO(300 nm)/STO, PSMO/STO single layer, PSMO bulk, and PCMO/STO single layer thin film.

H (T)	T_MIT_				
	PSMO/PCMO/STO annealed	PSMO(300 nm)/PCMO(300 nm)/STO^29^	PCMO/STO	PSMO/STO^12^	PSMO bulk^12^
0	215 K	231 K	Insulating	81 K	226 K
5	245 K	250 K	Insulating	105 K	230 K
10	280 K	270 K	80 K	125 K	240 K

To comprehend the electrical conduction mechanism in the grown heterostructure, the obtained temperature-dependent resistance curves were fit with different models. The high-temperature insulating regime was fit with the conduction mechanism pertaining to the adiabatic small polaron hopping model (ASPHM) given by Eq. (1), where, A is a constant of proportionality, E_A_ is the activation energy, k_B_ is the Boltzmann constant, and T is the absolute temperature^12,29,33^.

1 $$R = ATexp\left( {E_{A} /k_{B} T} \right)$$

The value of the hopping energy, E_A_ obtained for the heterostructure is found to be 109 meV at 0 T. The obtained E_A_ values are comparable to that obtained for PSMO/STO single layer (E_A_ = 120 meV)^12^. Thus, we observe that the values of the hopping energy of the conduction electrons in the high-temperature insulating regime are not influenced by the presence of the PCMO buffer layer or annealing conditions of the film. However, the obtained E_A_ values for the films are much higher than that obtained for the bulk PSMO (E_A_ = 42 meV)^12^. The conduction mechanism in the low-temperature regimes is usually explained in terms of the scattering contributions to the electrical resistance due to impurities, two-electron, two-magnon, electron-magnon, etc. In order to understand the conduction mechanism in the metallic regime, two models were used to fit the obtained data. For the metallic regime corresponding to T > 100 K, the two-magnon scattering mechanism, given by Eq. (2) yields better fit to the data, where R_0_ is the residual resistance contributed by the impurities, grain boundaries, and defects, R_2_ is the resistance due to two-electron scattering and R_4.5_ corresponds to the resistance due to two-magnon scattering mechanism^12,29,34^.

2 $$R = R_{0} + R_{2} T^{2} + R_{4.5} T^{4.5}$$

To understand the conduction mechanism in the regime corresponding to T < 100 K, the resistance curves were fit using Eq. (3), where, α is the residual resistance, β is the resistance corresponding to two-electron scattering, γ is the resistance due to inelastic scattering of magnons, and *n* gives the nature of inelastic scattering^35^.

3 $$R = \alpha - \beta \sqrt T + \gamma T^{n}$$

**Figure 7:**
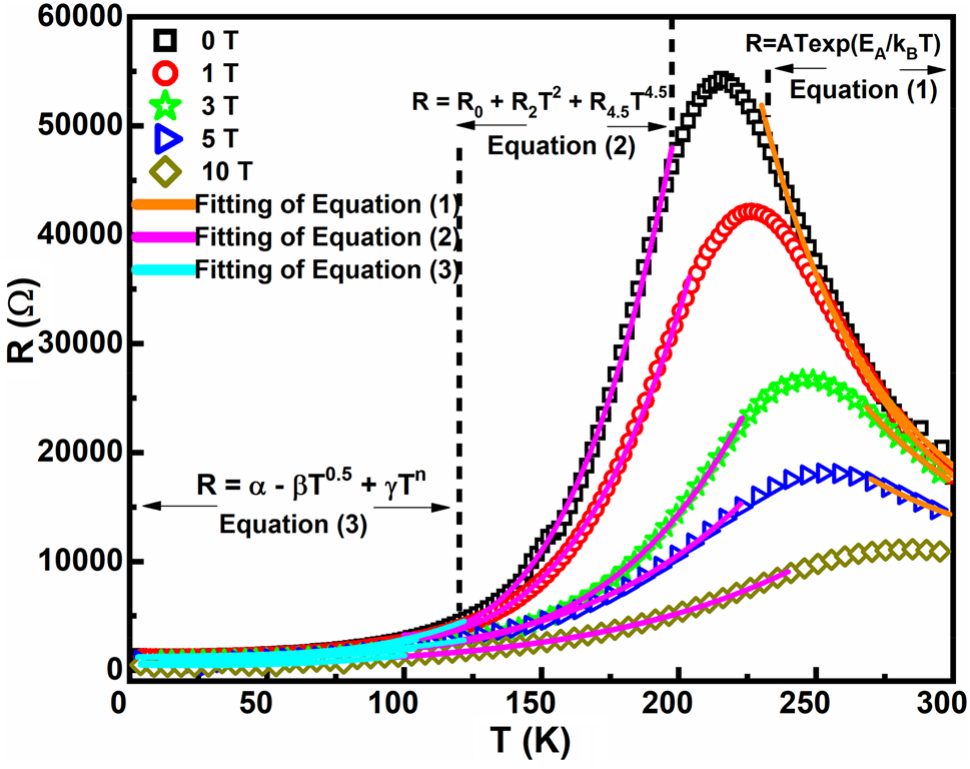
R(T) curve for different fields illustrating the fitting to understand the electrical conduction mechanism in the three different regimes.

The value of *n* obtained for the ultra-thin heterostructure is nearly 3, which corresponds to the one-magnon scattering mechanism^35^. The obtained *n* value shows that for T < 100 K, the one-magnon scattering mechanism is prevalent in the heterostructure. However, the value of *n* obtained in our previous studies on PSMO^12,29^ for T < 100 K was 4.5, indicating the dominant scattering mechanism to be due to two-magnons. This deviation in the scattering mechanism for T < 100 K in the ultra-thin heterostructure from two-magnon to one-magnon scattering further substantiates the idea that the FM ordering has strengthened in the ultra-thin heterostructure due to the melting of the AFM states in the PCMO. The resistance curves and the fitting in the three regimes are shown in Fig. 7.

### Cooling and heating in unequal fields

**Figure 8:**
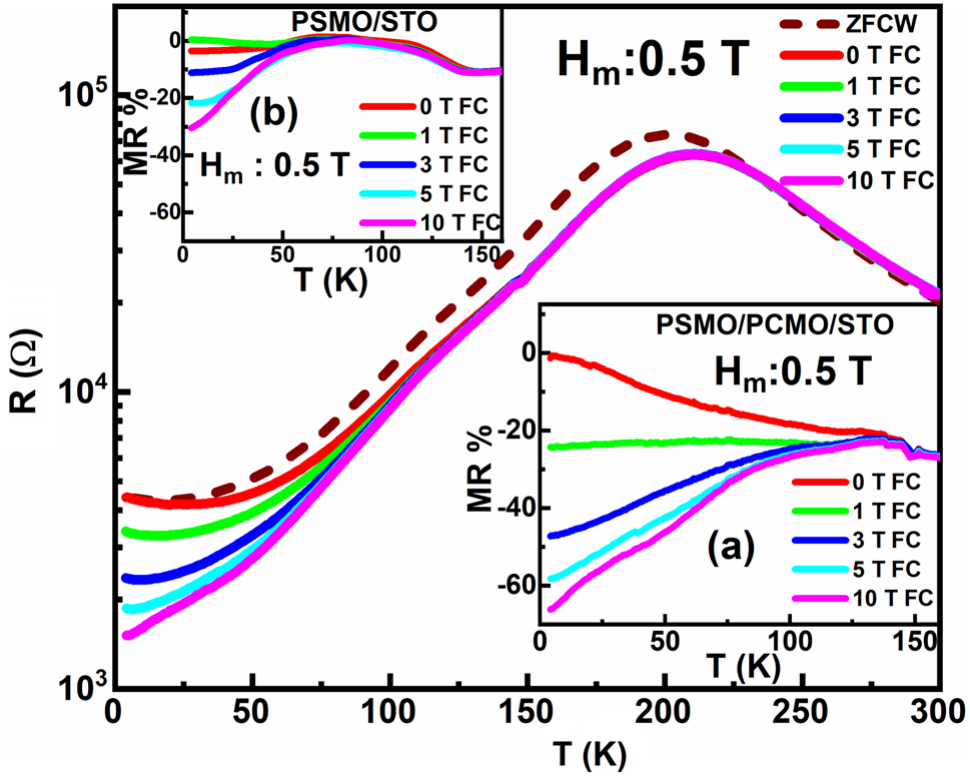
Cooling and heating under unequal field measurement protocol for PSMO/PCMO/STO heterostructure for H_m_ = 0.5 T. The inset (**a**) shows the MR% of the kinetically arrested FM phase in the heterostructure. The inset (**b**) shows similar measurements on annealed PSMO/STO thin film.

Cooling and heating in unequal fields (CHUF) is a special measurement protocol used to identify the kinetically arrested phases in a phase-segregated system^35–39^. In this CHUF protocol, the heterostructure is warmed from 4 to 300 K in a fixed measuring field (H_m_ = 0.5 T) after cooling it from 300 to 4 K under various applied magnetic fields, H_a_. The magnetic field switching from H_a_ to H_m_ is done at 4 K. The results obtained from the CHUF protocol measurements are illustrated in Fig. 8. It can be seen that though the insulating state is not affected in these curves, the metallic state below 150 K is highly modified by the value of H_a_ used for cooling the sample. For T ≤ 150 K, in the metallic state, the same H_m_ gives different curves for different H_a_. To understand the thermal evolution of MR due to CHUF in the heterostructure, MR % is calculated^35^ using Eq. (4), where R(0) and R(H) are the resistance values recorded in the zero-field cooled warming cycle, and H_a_ (= 1 T, 3 T, 5 T, and 10 T) cooled H_m_ (= 0.5 T) warming cycle, respectively.

4 $$MR\% = \frac{{\left( {R\left( H \right) - R\left( 0 \right)} \right)}}{R\left( 0 \right)} \times 100$$

The obtained curves of MR% are shown in the inset (a) of Fig. 8. For the same H_m_ = 0.5 T, with an increase in the value of H_a_, the value of resistance is found to drop drastically, which is reciprocated as an increase in the negative MR %. The obtained MR % is a measure of the volume fraction of FM state getting kinetically arrested, as shown in the inset (a) of Fig. 8. The observed behaviour confirms the phase-segregated nature of the ultra-thin heterostructure, wherein different amounts of the FM phases are getting kinetically arrested in each of these curves. This observation of kinetic arrest can be explained by considering the magnetic transitions of PCMO around 150 K into the AFM state. Below T = 150 K, while PSMO is in the FM state, PCMO undergoes a magnetic transition from CO state to an AFM state. Hence, for temperatures below 150 K, the FM phase of PSMO coexists with the AFM phase of PCMO. With increasing H_a_, the AFM state of PCMO melts into the FM state, as can be perceived from the increasing volume fraction of the FM phase that gets kinetically arrested at higher H_a_ by suppressing the AFM state of PCMO. Usually, very high fields are required to melt the AFM state of PCMO into the FM state. It is speculated that the increasing strength of the magnetic double-exchange interaction in PSMO with increasing H_a_ can provide the additional magnetic field required to melt the AFM state of PCMO. As a result, the AFM state of PCMO melts at a lower H_a_ into the FM state, thereby increasing the volume of the FM phase in the system. Thus, it is ascertained that the proximity of the FM PSMO has an influence on inducing the FM phase in the PCMO by triggering the melting of the AFM state at much lower values of H_a_ in PCMO. A similar measurement protocol adopted in the resistivity measurement by Krichene et al*.*^35^ has resulted in the kinetic arrest of different amounts of the FM phase in a phase segregated CO manganite La_0.4_Gd_0.1_Ca_0.5_MnO_3_, where the FM phase from the sample and the CO-AFM state from the parent compound coexist. However, in the present study, the possibility of the origin of the kinetically arrested state arising exclusively from PSMO can be neglected as the kinetic arrest in its annealed single layer counterpart PSMO/STO is much lower, as perceived from the inset (b) of Fig. 8. Hence, the volume of the kinetically arrested phase has increased remarkably due to the contribution from the melting of the AFM states present in the PCMO layer.

**Figure 9:**
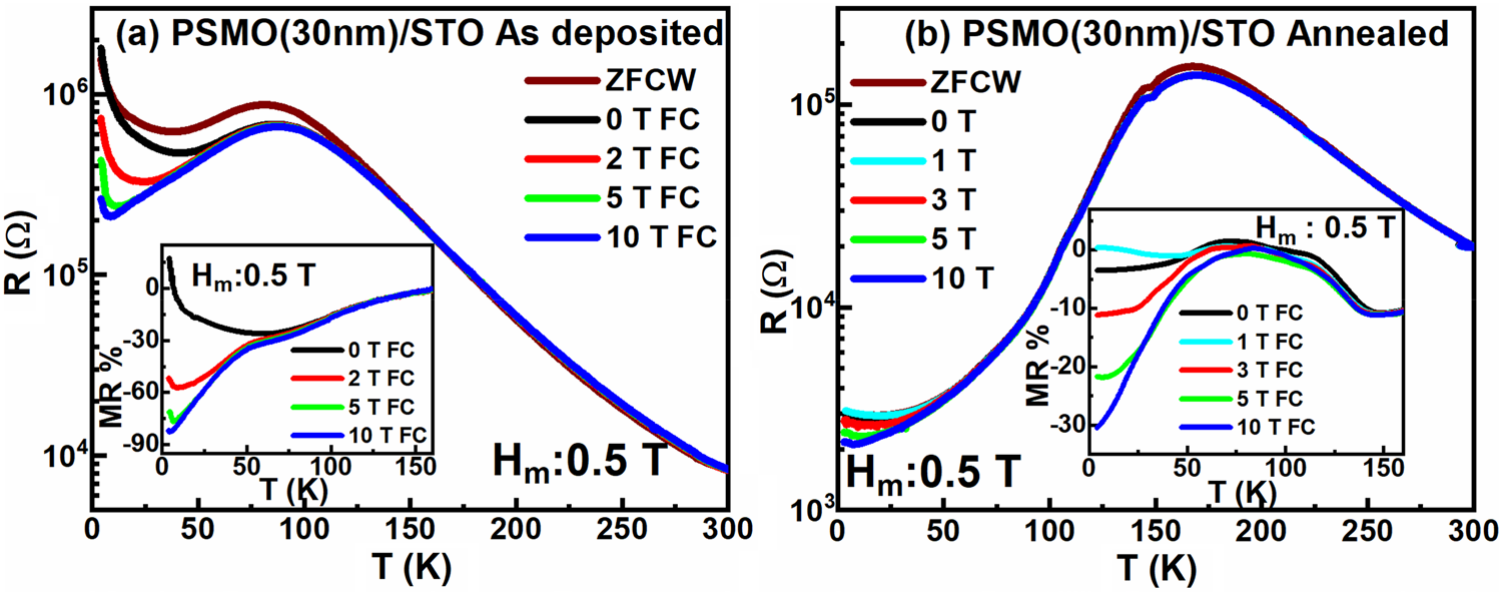
Cooling and heating under unequal field measurement protocol of PSMO(30 nm)/STO film for H_m_ = 0.5 T (**a**) before and (**b**) after ex-situ annealing. The insets show the MR% of the kinetically arrested FM phase in the film.

A comparative study was carried out using the CHUF protocol measurements on the PSMO single layer thin film before and after ex-situ annealing to elucidate the possibility of the origin of kinetic arrest due to inhomogeneity in the individual layer. The results obtained from the CHUF protocol measurements are illustrated in Fig. 9a and b, for the single layer PSMO thin film before and after ex-situ annealing, respectively. The obtained curves of MR % for PSMO film before and after ex-situ annealing are shown in the insets of Fig. 9a and b, respectively. The observed behaviour confirms the phase-segregated nature of the films, wherein the FM phase of the PSMO coexists with the AFM phases (from the parent compound) arising due to the inhomogeneity in the sample. A considerable change in the MR % with increasing H_a_ is observed in the as-deposited PSMO film, as shown in the inset of Fig. 9a. However, the kinetic arrest in its annealed counterpart is much lower, as perceived from the inset of Fig. 9b. Hence, it is concluded that the ex-situ annealing has enabled the annihilation of the AFM phases present in the phase-segregated as-deposited PSMO film into FM phases, thereby increasing the volume of the FM phase content in the sample and making the film more homogeneous. Hence, the kinetic arrest and training effect observed in the ultra-thin heterostructure of PSMO and PCMO do not arise due to inhomogeneity in individual (PSMO or PCMO) layers but due to the proximity of the two competing magnetic ground states (FM state of PSMO and CO-AFM state of PCMO).

**Figure 10:**
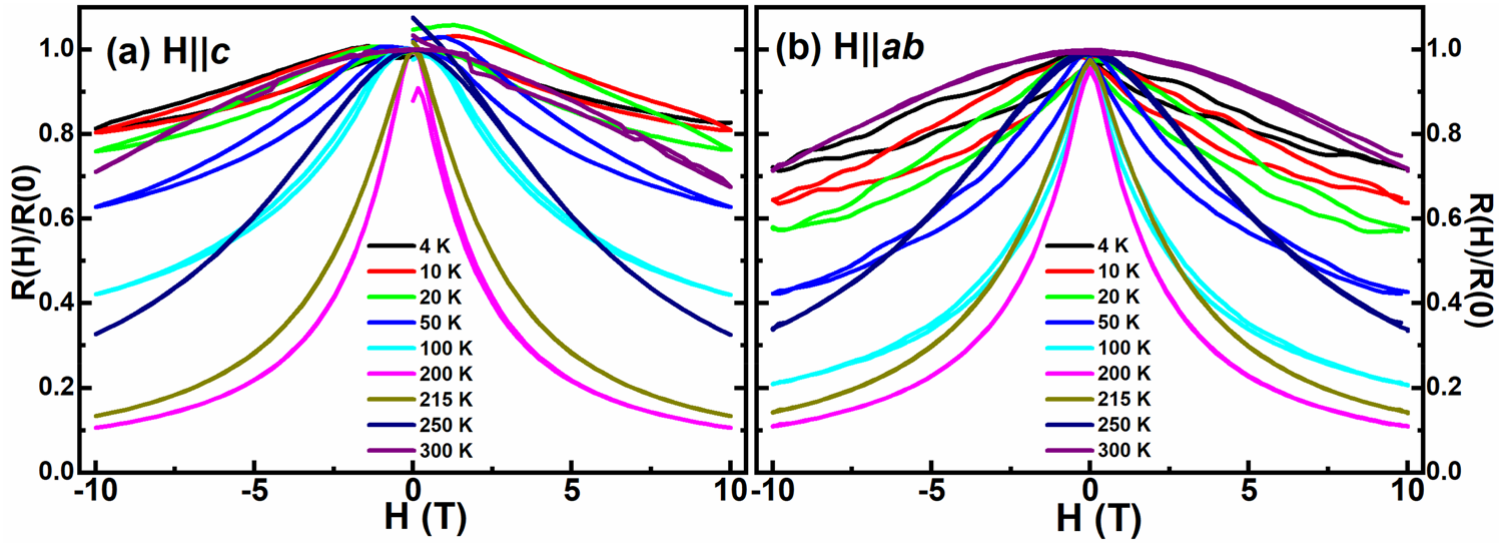
Isothermal evolution of the resistance as a function of the applied magnetic field obtained for the ultra-thin heterostructure in (**a**) H||c and (**b**) H||ab configurations.

**Figure 11:**
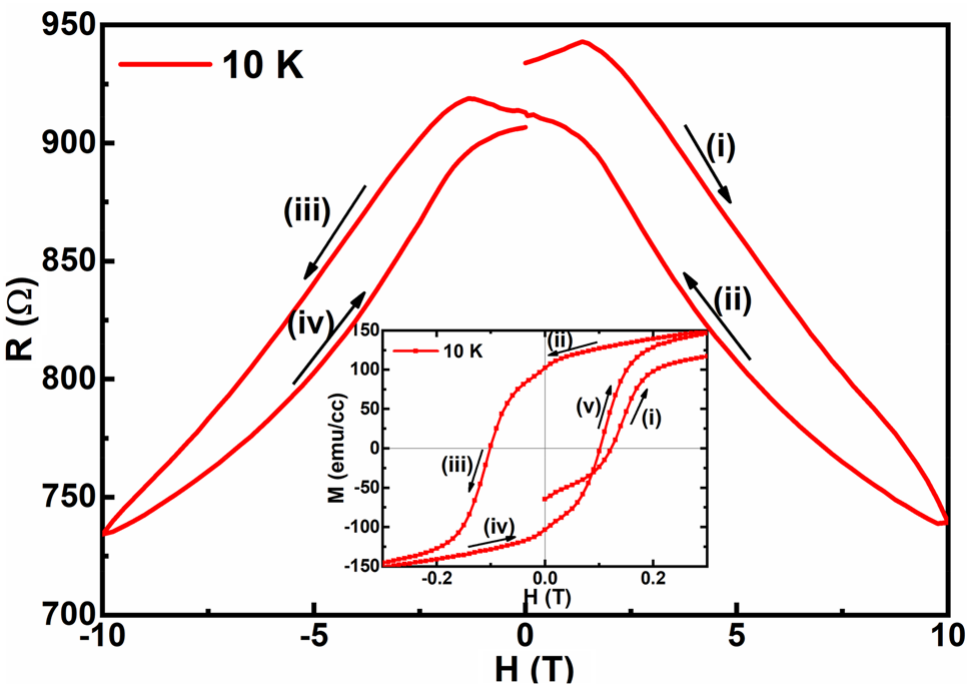
Training effect observed in the isothermal field-dependent resistance measurement for PSMO/PCMO/STO. Inset shows a similar observation in the isothermal field-dependent magnetization measurements for PSMO/PCMO/STO.

Figures 10a and b represent the isothermal evolution of resistance as a function of the applied magnetic field (R(H)) obtained for the ultra-thin heterostructure in H||*c* and H||*ab* configurations, respectively. Interestingly, a hysteresis is present in these curves even up to 10 T for T < 200 K, which confirms the phase-segregated nature of the heterostructure in both the configurations, as shown in Fig. 10a and b. Thus, the AFM state arising from the PCMO and the FM state from the PSMO coexist in the phase-segregated state. Though some of the AFM states of the PCMO melt into the FM state due to the applied magnetic field, which is further enhanced by the proximity of PSMO, the AFM state is not completely annihilated to the FM state and hence is present in small quantities even at the lowest temperature. For each configuration, the resistivity was recorded in four steps as follows: (i) from 0 to 10 T, (ii) from 10 to 0 T, (iii) from 0 to − 10 T, (iv) from − 10 to 0 T, as illustrated in Fig. 11. The resistances of the sample before step (i) and after step (iv), though expected to be the same, were found to be different. Hence, the MR response of the ultra-thin heterostructure depends on the applied magnetic field cycle, with its resistance decreasing with the H sweeps. Similar observation can also be seen in the M-H curve of the heterostructure as shown in the inset of Fig. 11, where the magnetization was recorded in five steps as follows: (i) from 0 to 7 T, (ii) from 7 to 0 T, (iii) from 0 to − 7 T, (iv) from − 7 to 0 T, and (v) from 0 to 7 T. An increased magnetic moment after training the sample through the magnetic fields was observed, as can be perceived from the obtained difference in the values of the magnetic moment for curves (i) and (v), as shown in the inset of Fig. 11. This behaviour can be attributed to the training effect^40^, wherein by training the sample through different magnetic field cycles, the volume of the FM metallic phase is increased. This leads to a decrease in the resistance in the R(H) curve by favouring the conduction of e_g_ electrons and an increase in the magnetic moment in the M-H curve by strengthening the double exchange mechanism. The hysteresis in the R(H) curve is observed for T < 200 K with the area of the hysteresis loop increasing with a further decrease in the temperature and is absent for T > 200 K. The above mentioned observation confirms that the observed training effect by the application of the magnetic field is due to more AFM states getting annihilated to the FM state with increasing field sweeps in the range T < 200 K. The formation of the FM phase from the AFM state of PCMO is further found to be enhanced at lower temperatures. Also, it is observed that the hysteresis curve is not symmetric, i.e., the resistances in the curve (i) and (iii) are higher than that of (ii) and (iv). In steps (i) and (iii), the sample is taken from 0 to 10 T, and the ground state of PCMO is the AFM state. As H approaches 10 T, the AFM state melts, leading to the formation of more FM states, creating a conduction path for the e_g_ electrons. Nevertheless, the ground state being AFM, the e_g_ electrons will encounter resistance to their percolation, yielding slightly higher resistance values for these curves^35^. However, in steps (ii) and (iv), where the sample is swept from 10 to 0 T, the ground state of PCMO has a predominantly FM state coexisting with the remnant AFM states. The ground state being predominantly FM may provide better conduction paths for the e_g_ electrons, resulting in a lower resistance for the corresponding curves.

**Figure 12:**
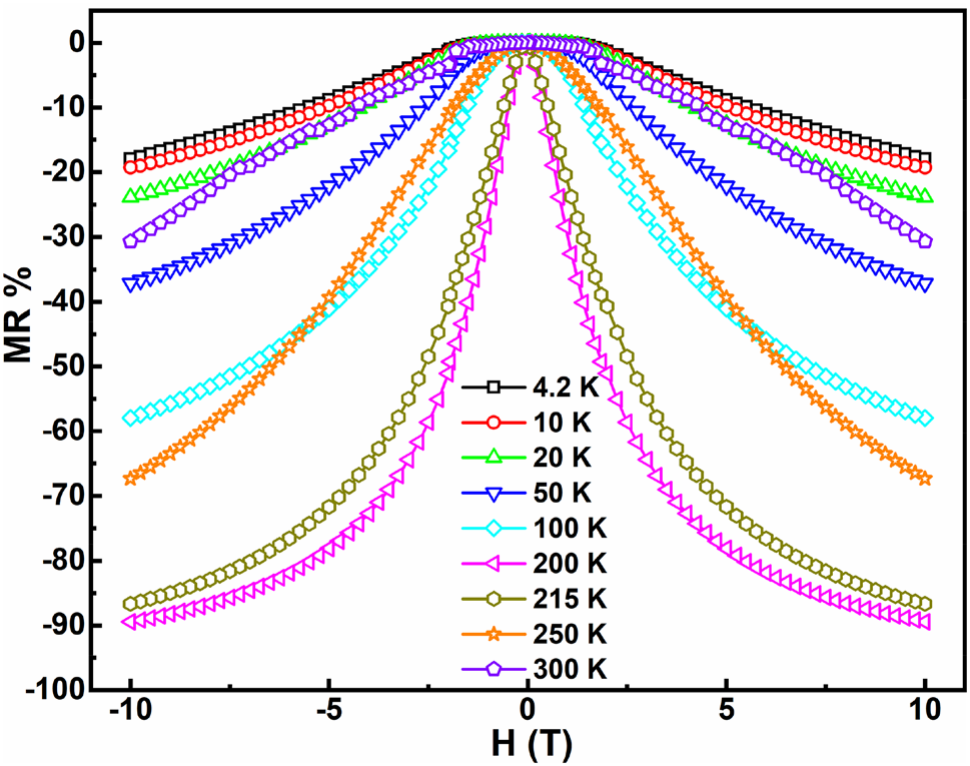
MR % obtained from magnetotransport measurement for PSMO/PCMO/STO ultra-thin heterostructure at different temperatures in the magnetic field range of ± 10 T.

The percentage response of magnetoresistance (MR %) of the ultra-thin heterostructure is represented in Fig. 12. The MR % is calculated^29^ for each isotherm using Eq. (4), where R(0) and R(H) are the resistance of the ultra-thin heterostructure obtained at 0 T and an applied magnetic field ‘H’, respectively. The heterostructure exhibits a maximum negative MR % of nearly 90% at 200 K and 10 T, which is observed to be near its T_MIT_. The values of MR % obtained for the ultra-thin heterostructure for temperatures near to room temperature are tabulated in Table 2, along with the corresponding values obtained for our previously studied samples for comparison. From Table 2, it can be seen that the MR % is enhanced in the ultra-thin heterostructure in the temperature range of 200–300 K compared to the thicker heterostructure^29^, the single-layer PSMO^12^ and the bulk PSMO^12^. At room temperature (300 K), MR % is found to be as high as 31% in the case of the ultra-thin heterostructure. Around 200 K i.e., near its T_MIT_, the heterostructure can be used for low-field CMR based applications. Near its T_MIT_, the heterostructure exhibits a negative MR % of nearly 90% at 200 K and 10 T, which becomes ~ 80% and ~ 55% at 5 T and 2 T, respectively. Enhanced CMR properties near T_MIT_ have been reported in the heterostructural superlattices of manganites^24–26^. Our previous report on the thicker heterostructure of PSMO and PCMO exhibited enhanced CMR ~ 20% near room temperature^29^. However, in the present study, we have been successful in further enhancing the MR % near room temperature by adjusting the thickness of the individual layers and ex-situ annealing. Achieving high values of MR % ~ 31% at room temperature in the PSMO/PCMO/STO ultra-thin heterostructure makes it one of the best candidates for high-field CMR-based applications like memory storage devices, spintronic devices, etc., near room temperature. We believe that further tailoring of the thicknesses of the individual layers or the annealing conditions of the heterostructure may facilitate in achieving this enhanced CMR near room temperature at much lower applied magnetic fields.

**Table 2:** Comparison of the MR % of ultra-thin PSMO/PCMO/STO heterostructure with its thicker heterostructure (PSMO(300 nm)/PCMO(300 nm)/STO), PSMO/STO single layer, and PSMO bulk.

Temperature (K)	( −) MR % at 10 T			
	PSMO/PCMO/STO	PSMO(300 nm)/PCMO(300 nm)/STO^29^	PSMO/STO^12^	PSMO bulk^12^
200	90	63	69	42
230	82	57	53	39
250	67	51	41	36
270	56	37	30	35
300	31	20	12	34

## Conclusion

In the present work, the ultra-thin heterostructure of PSMO/PCMO/STO was fabricated using the pulsed laser deposition technique. The structural characterization confirmed the presence of PSMO and PCMO layers in the heterostructure with high *c*-axis orientation. The morphological analysis illustrated the growth of the rod-shaped nano-grains embedded in a smooth matrix of the heterostructure. Magnetization measurements performed on the heterostructure exhibited the phase-segregated nature wherein the ferromagnetism of PSMO and the AFM state of PCMO coexist in proximity. These AFM states of PCMO coexisting with the FM state of PSMO were found to subsequently melt due to the interfacial interaction arising from the magnetic proximity into FM states. The signatures of the proximity effects were also observed in the magnetotransport measurements. Due to the phase-segregated nature of the grown ultra-thin heterostructure, it was found to exhibit two interesting phenomena, namely, training effect and kinetic arrest. The T_MIT_ was found to shift towards higher temperatures due to the proximity effect related magnetic exchange interaction of the FM clusters at the interface of the heterostructure. The electrical conduction in the high-temperature regime was studied in terms of the hopping of the adiabatic small polarons. In addition to the resistance contributed by the impurities and electron–electron scattering mechanism, the scattering due to magnons also contributed towards resistance in the metallic regime, which was found to be of two different origins for T > 100 K and T < 100 K. While the resistance in the metallic regime for T > 100 K was found to be contributed by the inelastic scattering due to two-magnons, in the metallic regime below 100 K, the one-magnon inelastic scattering contribution was found to be prevalent. The heterostructure was also found to possess magnetic proximity-driven interfacial interaction resulting in an enhanced colossal magnetoresistance property near room temperature, making it a good candidate for CMR-based technological applications near room temperature.

## Methods

**Figure 13:**
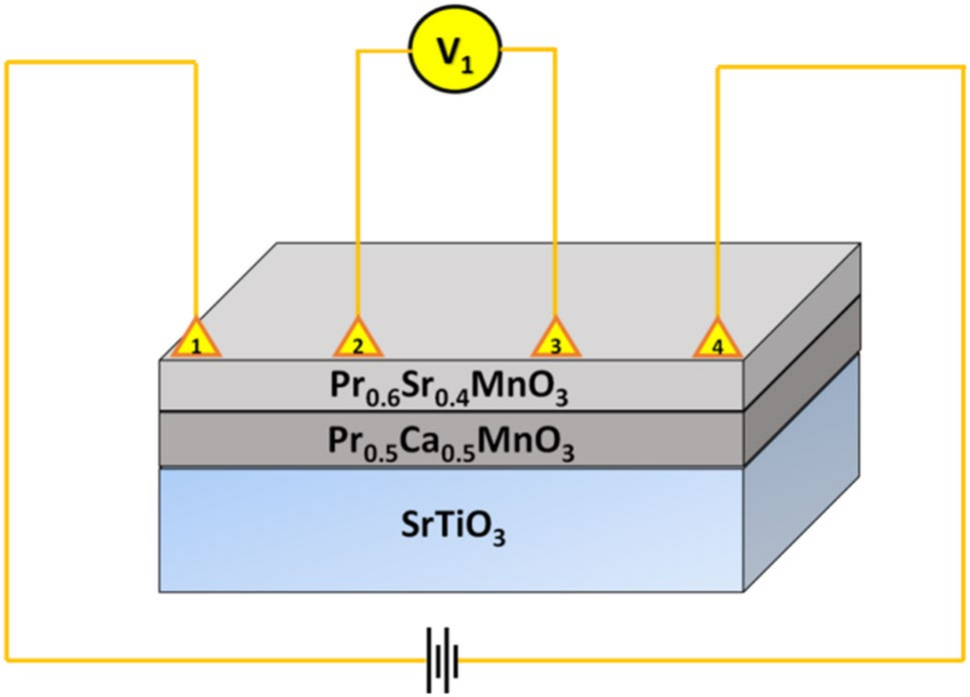
Schematic representation of the heterostructure in linear geometry for magnetoresistance measurement.

The heterostructural thin film of Pr_0.6_Sr_0.4_MnO_3_ (PSMO) and Pr_0.5_Ca_0.5_MnO_3_ (PCMO) was fabricated on a single crystalline (1 0 0) oriented substrate of SrTiO_3_ (STO) using the pulsed laser deposition technique. The targets used for the deposition were synthesized using the standard solid-state reaction method. The experimental conditions used to synthesize the targets were reported elsewhere^12,13^. Initially, 15 nm of the PCMO was deposited on STO, followed by the deposition of 15 nm of PSMO. It is reported that nearly 12 nm to 15 nm are required to gradually change the in-plane lattice parameter of the manganite from that of the substrate to that of the manganite (for PSMO = 3.850 Å)^41^. Hence, the ideal minimum thickness required for such studies will be 15 nm of the individual layer. Also, the proximity effects and the interfacial interactions become more prominent in the ultra-thin heterostructures than the thicker heterostructures. In thicker heterostructures these proximity effects may not be distinguishable as the bulk properties of the corresponding layers will be dominating as seen in our previous study on the thicker heterostructure^29^. Hence, we chose 30 nm (15 nm + 15 nm) heterostructure for the present study. The optimized growth conditions used for the deposition of the individual layers of PCMO and PSMO on STO substrate were reported in our earlier studies^12,13,29^. Further, the heterostructure was annealed at 840 °C for 24 h in air. The structural characterization of the grown heterostructural film was performed in the Engineering applications beamline (BL-2), Indus-2 Synchrotron facility, RRCAT, Indore (India)^42^. X-rays of wavelength 0.826 Å were utilised for the θ−2θ powder X-ray diffraction (XRD), grazing-incidence X-ray diffraction (GI-XRD), and high-resolution X-ray diffraction (HR-XRD) measurements. Data was collected using the Dectris detector (MYTHEN2 X 1 K) in the reflection geometry. The morphology and the elemental analyses of the fabricated heterostructure were performed using scanning electron microscopy (SEM) combined with energy-dispersive X-ray spectroscopy (EDS). The thickness of the grown heterostructure was confirmed by fracture cross-sectional SEM imaging of the sample. A field emission scanning electron microscope (FE-SEM) with 30 kV acceleration voltage, model SUPRA 55 by Carl Zeiss, Germany was used. A 10 mm^2^ liquid nitrogen free Silicon drift detector (SDD) by Oxford Instruments Inc. (model: X-act) attached to the SEM was used for EDS analysis (by INCA EDS software). Magnetization measurements were performed in the temperature range of 4–300 K and in the applied magnetic field range of 0 to ± 7 T, applied parallel (H||*ab*) to the plane of the heterostructure, using a Quantum Design Ever-Cool SQUID magnetometer. Zero-field cooled (ZFC) and field cooled (FC) data correspond to the magnetic moments recorded for the sample in the warming cycle in an applied magnetic field (H), after cooling the sample to the lowest temperature in zero field and the applied field (H), respectively. The substrate contribution towards the magnetization data was eliminated by repeating the measurements in identical conditions on a bare substrate of the same dimension and subtracting it from the obtained data of the heterostructure. Magnetotransport measurements were performed in the linear geometry, using a 15 T cryofree MR system from Cryogenic, UK, in the temperature range of 4–300 K and the magnetic field range of 0 to ± 15 T, applied both parallel (H||*ab*) and perpendicular (H||*c*) to the plane of the heterostructure. Figure 13 represents the schematic diagram of the heterostructure in the linear geometry, where the current is passed across leads 1 and 4, and the corresponding voltage drop is measured across leads 2 and 3. The special measurement protocol of cooling and heating in unequal fields (CHUF) was performed to investigate the coexistence of the different magnetic orderings.

## Data Availability

The datasets used and/or analysed during the current study are available from the corresponding author on reasonable request.
